# Modeling Individual Patient Count/Rate Data over Time with Applications to Cancer Pain Flares and Cancer Pain Medication Usage

**DOI:** 10.4236/ojs.2021.115038

**Published:** 2021-09-30

**Authors:** George J. Knafl, Salimah H. Meghani

**Affiliations:** 1 School of Nursing, University of North Carolina at Chapel Hill, Chapel Hill, NC, USA; 2 Department of Biobehavioral Health Sciences, School of Nursing, University of Pennsylvania, Philadelphia, PA, USA

**Keywords:** Adaptive Regression, Extended Linear Mixed Modeling, Generalized Estimating Equations, Likelihood-Like Cross-Validation, Poisson Regression

## Abstract

The purpose of this article is to investigate approaches for modeling individual patient count/rate data over time accounting for temporal correlation and non-constant dispersions while requiring reasonable amounts of time to search over alternative models for those data. This research addresses formulations for two approaches for extending generalized estimating equations (GEE) modeling. These approaches use a likelihood-like function based on the multivariate normal density. The first approach augments standard GEE equations to include equations for estimation of dispersion parameters. The second approach is based on estimating equations determined by partial derivatives of the likelihood-like function with respect to all model parameters and so extends linear mixed modeling. Three correlation structures are considered including independent, exchangeable, and spatial autoregressive of order 1 correlations. The likelihood-like function is used to formulate a likelihood-like cross-validation (LCV) score for use in evaluating models. Example analyses are presented using these two modeling approaches applied to three data sets of counts/rates over time for individual cancer patients including pain flares per day, as needed pain medications taken per day, and around the clock pain medications taken per day per dose. Means and dispersions are modeled as possibly nonlinear functions of time using adaptive regression modeling methods to search through alternative models compared using LCV scores. The results of these analyses demonstrate that extended linear mixed modeling is preferable for modeling individual patient count/rate data over time, because in example analyses, it either generates better LCV scores or more parsimonious models and requires substantially less time.

## 1. Introduction

An ongoing study (NIH/NINR 1R01NR017853) of patients with cancer is collecting daily longitudinal count/rate data including numbers of pain flares per day and numbers of as needed pain medications taken per day. Data are being collected for each study participant over periods of up to five months long. A completed study (NIH/NINR RC1NR011591) collected numbers for cancer patients over 3 months of around the clock pain medications taken per day per dose, that is, the number of times a medication is taken in a day relative to the number of doses that are supposed to be taken in a day. Standard assumptions of means linear in time and dispersions constant over time are not always appropriate for such data. Also, a Poisson process assumption of independence over time needs not always hold. A model selection score needs to be defined for evaluating models for the data and for use in searches over alternative models. Times to conduct these searches need to be as short as possible, especially as the number of time measurements increases.

Approaches are presented for modeling mean counts/rates over time separately for each individual patient controlling for temporal correlation as well as for time-varying dispersions. These approaches use Poisson regression methods, because count/rate data are being modeled. Generalized estimating equations (GEE) methods [1] [2] provide a natural choice for modeling correlations for such count variables. However, standard GEE methods have limited value, because they assume constant dispersions. Furthermore, GEE methods avoid specification of likelihood functions, which are useful for generating model selection criteria. In what follows, two extensions of GEE methods are formulated and evaluated that address temporal correlation and time-varying means and dispersions for repeated count/rate measurements. A likelihood-like function, that is, a function used like a likelihood but which needs not integrate to 1, is defined and used in computing parameter estimates for these extensions along with a model selection criterion for comparing alternative models. Example analyses of selected individual cancer patient count/rate data are presented using adaptive regression methods [3] for identifying possibly nonlinear trajectories for means and dispersions of counts/rates over time while controlling for temporal correlation.

## 2. Modeling Individual Count/Rate Data

### 2.1. Generalized Linear Modeling of Means

Let *y*_*t*(*i*)_ denote count values for an individual patient observed at *N* distinct times within a general set *T* of times, that is, *t*(*i*) ∈ *T* = {*t*(*i*):1 ≤ *i* ≤ *N*}. Combine these into the *N* × 1 vector ***y***. Let *μ*_*t*(*i*)_ = *Ey*_*t*(*i*)_ denote associated mean or expected counts and combine these into the *N* × 1 vector ***μ***. Denote the residuals as *e*_*t*(*i*)_ = *y*_*t*(*i*)_ − *μ*_*t*(*i*)_ for *t*(*i*) ∈ *T* and combine these into the *N* × 1 vector ***e*** = ***y*** – ***μ***. Let *x*_*t*(*i*), *j*_ denote predictor values over times *t*(*i*) ∈ *T* and over predictors indexed by 1 ≤ *j* ≤ *J* and combine these into the *J* × 1 vector ***x***_*t*(*i*)_ with transpose denoted by $x_{t(i)}^{\text{T}}$ for *t*(*i*) ∈ *T*. Let **X** be the *N* × *J* matrix with rows $x_{t(i)}^{\text{T}}$ for 1 ≤ *i* ≤ *N*. Let ***β*** denote the associated *J* × 1 vector of coefficient parameters. Use generalized linear models [4] [5] of *μ*_*t*(*i*)_ for *t*(*i*) ∈ *T* with natural log link function *h*(*μ*) = log_*e*_ (*μ*) so that $h\left(\mu_{t(i)}\right)=x_{t(i)}^{\text{T}}\cdot \beta$ for *t*(*i*) ∈ *T*. When *x*_*t*(*i*),1_ = 1 for *t*(*i*) ∈ *T*, the first entry *β*_1_ of ***β*** is an intercept parameter. Treat each *y*_*t*(*i*)_ as Poisson distributed so that its variance is *V* (*μ*_*t*(*i*)_) = *μ*_*t*(*i*)_.

The counts *y*_*t*(*i*)_ sometimes have associated totals *Y*_*t*(*i*)_ > 0, and then the model for the mean counts *μ*_*t*(*i*)_ is converted to a model for the means $\mu_{t(i)}^{\prime }$ of the rates $y_{t(i)}^{\prime }=y_{t(i)}/Y_{t(i)}$ using offsets *o*_*t*(*i*)_ = log(*Y*_*t*(*i*)_). Formally, replace $x_{t(i)}^{\text{T}}\cdot \beta$ by $x_{t(i)}^{\text{T}}\cdot \beta +o_{t(i)}$ so that mean counts are $\mu_{t(i)}=\text{exp}\left(x_{t(i)}^{\text{T}}\cdot \beta +o_{t(i)}\right)$ and then

$$\mu_{t(i)}^{\prime }=Ey_{t(i)}^{\prime }=\mu_{t(i)}/Y_{t(i)}=\text{exp}\left(x_{t(i)}^{\text{T}}\cdot \beta \right)$$

are the mean rates.

### 2.2. Time-Varying Dispersions

Let $x_{t(i),j}^{\prime }$ denote predictor values over times *t*(*i*) ∈ *T* and over predictors indexed by 1 ≤ *j* ≤ *J*′ and combine these into the *J*′ × 1 vectors $x_{t(i)}^{\prime }$ for *t*(*i*) ∈ *T*. Let **X**′ be the *N* × *J*′ matrix with rows $x_{t(i)}^{\prime \text{T}}$ for 1 ≤ *i* ≤ *N*. Let ***β***′ denote the associated *J*′ × 1 vector of coefficient parameters. Let *φ*_*t*(*i*)_ denote dispersion values over times *t*(*i*) ∈ *T* satisfying $\text{log}\left(\varphi_{t(i)}\right)=x_{t(i)}^{\prime \text{T}}\cdot \beta^{\prime }$ and define the extended variances as

$$\sigma_{t(i)}^{2}=\varphi_{t(i)}\cdot V\left(\mu_{t(i)}\right)=\varphi_{t(i)}\cdot \mu_{t(i)}$$

and the extended standard deviations as $\sigma_{t(i)}=\varphi_{t(i)}^{1/2}\cdot \mu_{t(i)}^{1/2}$ for *t*(*i*) ∈ *T*. Informally, these quantities extend the usual Poisson variances and standard deviations through multiplication by dispersions. These are used to compute the standardized residuals *stde*_*t*(*i*)_ = *e*_*t*(*i*)_/σ_*t*(*i*)._ Combine the extended standard deviations into the *N* × 1 vector *σ*. When $x_{t(i),1}^{\prime }=1$ for *t*(*i*) ∈ *T*, the first entry $\beta_{1}^{\prime }$ of ***β*′** is an intercept parameter. The constant dispersion model corresponds to $x_{t(i),1}^{\prime }=1$ for *t*(*i*) ∈ *T* with *J*′ = 1. This is the dispersion model used in standard GEE modeling.

When offsets *o*_*t*(*i*)_ are used to convert the model for the counts *y*_*t*(*i*)_ to a model for the rates $y_{t(i)}^{\prime }$, they can also be added to the dispersions. The dispersions then satisfy $\text{log}\left(\varphi_{t(i)}\right)=x_{t(i)}^{\prime \text{T}}\cdot \beta^{\prime }+o_{t(i)}$ so that the extended variances for the counts *y*_*t*(*i*)_ are

$$\sigma_{t(i)}^{2}=\varphi_{t(i)}\cdot \mu_{t(i)}=\text{exp}\left(x_{t(i)}^{\prime \text{T}}\cdot \beta^{\prime }\right)\cdot \text{exp}\left(x_{t(i)}^{\text{T}}\cdot \beta \right)\cdot {\text{exp}}^{2}\left(o_{t(i)}\right)$$

and then the variances for the rates $y_{t(i)}^{\prime }$ are

$$\sigma_{t(i)}^{\prime 2}=\sigma_{t(i)}^{2}/T_{t(i)}^{2}=\varphi_{t(i)}^{\prime }\cdot \mu_{t(i)}^{\prime }$$

where

$$\varphi_{t(i)}^{\prime }=\text{exp}\left(x_{t(i)}^{\prime \text{T}}\cdot \beta^{\prime }\right).$$

### 2.3. Modeling Correlations

Denote the covariance matrix for the count vector ***y*** as Σ. Use the GEE approach [1] [2] to model the covariance matrix for ***y*** as Σ = Diag(σ)·**R**(*ρ*)·Diag(***σ***) where Diag(***σ***) is the *N* × *N* diagonal matrix with diagonal entries σ_*t*(*i*)_ for *t*(*i*) ∈ *T* and **R**(*ρ*) is a *N* × *N* correlation matrix with diagonal entries 1 and off diagonal entries *R*_*t*(*i*),*t*(*i′*)_ for 1 ≤ *i* ≠ *i*′ ≤ *N* determined by a correlation parameter *ρ* varying with the assumed correlation structure. Under independent (IND) correlations, *R*_*t*(*i*),*t*(*i′*)_ = *ρ*_IND_ = 0 for 1 ≤ *i* ≠ *i*′ ≤ *N*. A Poisson process generates such correlations. Under exchangeable (EXCH) correlations, *R*_*t*(*i*),*t*(*i′*)_ = *ρ*_EXCH_ for 1 ≤ *i* ≠ *i*′ ≤ *N*, that is, the correlations are constant. Under autoregressive of order 1 (AR1) correlations, $R_{t(i),t\left(i^{\prime }\right)}=\rho_{\text{AR}1}^{\left|t(i)-t\left(i^{\prime }\right)\right|}$ for 1 ≤ *i* ≠ *i*′ ≤ *N* where |*t*(*i*) − *t*(*i′*)| is the absolute value of *t*(*i*) − *t*(*i′*). These differences are all assumed to be integers so that the correlations *R*_*t*(*i*),*t*(*i′*)_ are all well-defined. In general, *R*_*t*(*i*),*t*(*i′*)_ are spatial AR1 correlations. The special case of non-spatial AR1 correlations with *t*(*i*) = *i* treats times as equally spaced. The parameter *ρ*_AR1_ is called the autocorrelation.

### 2.4. Possible Extensions

The above formulation can be extended to address repeated measurements of types other than counts/rates and for multiple patients. More complex correlation structures based on multiple correlation parameters can also be considered. One such example is unstructured correlations with different correlations for different pairs of measurements, but this requires data from multiple patients to be reasonably estimated. These extensions are not addressed further.

## 3. Standard Generalized Estimating Equations Modeling

### 3.1. Notation and Parameter Estimation

Under standard GEE modeling, dispersions are treated as a constant *φ*_0_ so that the covariance matrix satisfies

$$\Sigma =\varphi_{0}\cdot \text{Diag}\left(V^{1/2}(\mu )\right)\cdot R(\rho )\cdot \text{Diag}\left(V^{1/2}(\mu )\right)$$

where ***V***(***μ***) is the *N* × 1 vector with entries *V*(*μ*_*t*(*i*)_) = *μ*_*t*(*i*)_ for *t*(*i*) ∈ *T*. The generalized estimating equations are given by ***g***(***β***) = **0** where **0** is the *J* × 1 vector with all zero entries, ***g***(***β***) = **D**^T^ · Σ^−1^ · ***e***, and the *N* × *J* matrix **D** = ∂***μ/***∂***β*** with entries *D*_*t*(*i*),*j*_ = ∂*μ*_*t*(*i*)_ / ∂*β*_*j*_ = *x*_*t*(*i*),*j*_ · *μ*_*t*(*i*)_ for *t*(*i*) ∈ *T* and 1 ≤ *j* ≤ *J*. Let **H**(***β***) = − **D**^T^ · **Σ**^−1^ · **D**. Note that in the general GEE context with correlated outcomes for multiple subjects, the formulation for ***g***(***β***) would equal a sum of terms like **D**^T^ · **Σ**^−1^ · ***e*** for each subject and **H**(***β***) would equal a sum of terms like − **D**^T^ · **Σ**^−1^ · **D** for each subject. Only one such term is needed here since data for only one subject/patient are being modeled. The GEE process for estimating ***β*** iteratively solves ***g***(***β***) = 0 as follows. Given the current value ***β***_*u*_ for ***β***, the next value is given by ***β***_*u*+1_ = ***β***_*u*_ − **H**^−1^ (***β***_*u*_) · ***g***(***β***_*u*_), thereby adapting Newton’s method with ***g***(***β***) in the role of the gradient vector and **H**(***β***) in the role of the Hessian matrix.

The constant dispersion parameter *φ*_0_ is estimated using the Pearson residuals *Pe*_*t*(*i*)_ (***β***) = *e*_*t*(*i*)_/*V*^1/2^ (*μ*_*t*(*i*)_) evaluated at a given value for the mean coefficient parameter vector ***β***. The bias-adjusted estimate *φ*_0_(***β***) of the dispersion parameter *φ*_0_ satisfies $\varphi_{0}(\beta )=\sum_{i=1}^{N}Pe_{t(i)}^{2}(\beta )/(N-J)$ assuming *N* – *J >* 0. Next, the correlation parameter *ρ*(***β***) is estimated using standardized errors

$${\text{stde}}_{t(i)}(\beta )=\varphi_{0}^{-1/2}(\beta )\cdot Pe_{t(i)}(\beta )$$

for *t*(*i*) ∈ *T* as follows. The IND correlation structure has no need for an estimate. For the EXCH correlation structure and a given value ***β*** for the mean parameter vector, *ρ*_EXCH_ can be estimated by

$$\rho_{\text{EXCH}}(\beta )=\sum_{i=1}^{N-1}\sum_{i^{\prime }=i+1}^{N}{\text{stde}}_{t(i)}(\beta )\cdot {\text{stde}}_{t\left(i^{\prime }\right)}(\beta )/(N\cdot (N-1)/2-J)$$

assuming *N* · (*N −* 1)/2 − *J* > 0. For the AR1 correlation structure and a given value ***β*** for the mean parameter vector, the autocorrelation *ρ*_AR1_ can be estimated by

$$\rho_{\text{AR}1}(\beta )=\sum_{i=1}^{N-1}{\left({\text{stde}}_{t(i)}(\beta )\cdot {\text{stde}}_{t(i+1)}(\beta )\right)}^{1/(|t(i)-t(i+1)|)}/(N-1-J)$$

assuming *N −* 1 − *J* > 0. In the non-spatial AR1 special case,

$$\rho_{\text{AR}1}(\beta )=\sum_{i=1}^{N-1}\left({\text{stde}}_{t(i)}(\beta )\cdot {\text{stde}}_{t(i+1)}(\beta )\right)/(N-1-J)$$

because |*t*(*i*) − *t i*(+ 1)| = 1 for 1 ≤ *i < N*.

For any correlation structure, once the GEE estimate ***β***(*T*) of the coefficient parameter vector ***β*** is computed using the observations indexed by *t*(*i*) ∈ *T*, the GEE estimate of the dispersion parameter *φ*_0_ is *φ*_0_ (*T*) = *φ*_0_ (***β***(*T*)). The GEE estimate of the correlation parameter *ρ* is *ρ*(*T*) = *ρ*(***β***(*T*)) computed using ***β***(*T*) and *φ*_0_ (*T*).

### 3.2. The Likelihood-Like Function

Let ***θ*** = (***β***^T^φ_0_)^T^ be the (*J* + 1) × 1 vector of the GEE mean and dispersion parameters. The correlation parameter *ρ* is a function of ***β*** and *φ*_0_ and so has not been included in ***θ***. Use the multivariate normal likelihood to define the likelihood-like function *L*(*T*;***θ***) satisfying

$$\ell (T;\theta )=\text{log}(L(T;\theta ))=-e^{\text{T}}\cdot \Sigma^{-1}\cdot e/2-\text{log}(|\Sigma |)/2-N\cdot \text{log}(2\cdot \pi )/2$$

where |Σ| is the determinant of the covariance matrix **Σ**. The vector ∂*ℓ*(*T*;***θ***)/∂***β*** of partial derivatives of *ℓ*(*T*;***θ***) can be expressed as the sum of two terms. The first term corresponds to differentiating the residual vector part ***e*** of *ℓ*(*T*;***θ***) with respect to ***β*** holding the covariance part **Σ** fixed in ***β*** and equals ***g***(***β***), the gradient-like quantity used in standard GEE modeling. This fact seems to have been first recognized by Chaganty [6]. One advantage for having a likelihood-like function for GEE models is that it can be used to compute parameter estimates. Another is that it can be used to compute model selection criteria not otherwise available for GEE modeling.

### 3.3. Likelihood-Like Cross-Validation

Burman [7] defined *k*-fold cross-validation with observations partitioned into *k* disjoint subsets called folds. Fold observations are predicted using parameter estimates computed using the data from the other folds. In *k*-fold likelihood-like cross-validation (LCV), these deleted fold predictions are scored using the associated likelihood-like function *L*. Randomly partition the times *t*(*i*) ∈ *T* into *k* disjoint folds *T*(*f*) for 1 ≤ *f* ≤ *k*. Use the same initial seed for randomization with all models under consideration so that their LCV scores are comparable. Let ***θ***(*T\T*(*f*)) denote the estimate of ***θ*** using the data with times in the complement *T\T*(*f*) of the fold *T*(*f*). For 1 ≤ *f* ≤ *k*, let *T*^+^(*f*) denote the union of all folds *T*(*f*) for 1 ≤ *f* ′ ≤ *f* with *T*^+^(0) the empty fold and set *L*(*T*^+^(0);***θ***) = 1. Define the LCV score to satisfy

$$\text{LCV}=\prod_{f=1}^{k}{\text{LCV}}_{f}^{1/N}$$

where LCV_*f*_ is defined as the conditional likelihood-like term for the data in fold *T*(*f*) conditioned on the data in the union *T*^+^(*f − 1*) of the prior folds using the deleted estimate ***θ***(*T\T*(*f*)) of the parameter vector ***θ***. Formally,

$${\text{LCV}}_{f}=L\left(T(f)|T^{+}(f-1);\theta (T\backslash T(f))\right)\;=L\left(T^{+}(f);\theta (T\backslash T(f))\right)/L\left(T^{+}(f-1);\theta (T\backslash T(f))\right)$$

Because fold assignment is random, folds can be empty when the number *k* of folds is large relative to the number *N* of measurements, and then those folds are dropped from the computation of the LCV score. Larger LCV scores indicate better models. Note that even if the full data are non-spatial with observations at consecutive integer times *t*(*i*) = *i* for 1 ≤ *i ≤ N*, the folds *T*(*f*) and the fold unions *T*^+^(*f*) are not consecutive integer times except in rare cases and so require more general handling.

## 4. Incorporating Nonconstant Dispersions

### 4.1. Formulation

GEE modeling can be extended to handle nonconstant dispersions. Let ***θ*** = (***β***^T^ ***β***′^T^)^T^ be the (*J* + *J*′) × 1 vector of the mean and dispersion parameters. The definition of the likelihood-like function *L*(*T*;***θ***) given for standard GEE holds using the more general parameter vector ***θ***. Differentiate *ℓ*(*T*;***θ***) = log(*L*(*T*;***θ***)) with respect to the vector ***β***′ of dispersion coefficient parameters while holding the correlation parameter *ρ* fixed in the current parameter vector ***β***′ to provide the *J*′ estimating equations ***g***(***β***′) = ∂′*ℓ*(*T*;***θ***)/∂′***β***′ = **0** where the notation ∂′*ℓ*(*T*;***θ***)/∂′***β***′ is used to indicate that this is not the full partial derivative vector for *ℓ*(*T*;***θ***) in ***β***′ due to not accounting for the effect of ***β***′ on *ρ*. Now, combine these with the *J* standard GEE equations ***g***(***β***) = **0** to solve for joint estimates of ***β*** and ***β***′. Then, iteratively solve for

$$g(\theta )={\left(g{\left(\beta \right)}^{\text{T}}g{\left(\beta^{\prime }\right)}^{\text{T}}\right)}^{\text{T}}=0$$

with ***g***(***θ***) in the role of the gradient vector and the (*J* + *J*′) × (*J* + *J*′) matrix ***H***(***θ***) in the role of the Hessian matrix. ***H***(***θ***) has four component submatrices: the *J* × *J* matrix ***H***(***β***) for the mean coefficients as defined for standard GEE, the *J*′ × *J* ′ matrix ***H***(***β***′) = ∂′***g***(***β***′)/∂′***β***′ for the *J*′ dispersion coefficients, the *J* × *J*′ matrix ***H*** (***β***, ***β***′) = ∂′***g***(***β***)/∂′***β***′, and its transpose ***H*** (***β***′,***β***) = ***H***(***β***, ***β***′)^T^.

Note that

$$\begin{array}{l} {\text{log}}_{e}(|\Sigma |)={\text{log}}_{e}(|R(\rho )|)+\sum_{i=1}^{N}{\text{log}}_{e}\left(\varphi_{t(i)}\right)+\sum_{i=1}^{N}{\text{log}}_{e}\left(V\left(\mu_{t(i)}\right)\right),\\ \varphi_{t(i)}=\text{exp}\left(x_{t(i)}^{\prime \text{T}}\cdot \beta^{\prime }\right) \end{array}$$

and

$$e^{\text{T}}\cdot \Sigma^{-1}\cdot e={\text{stde}}^{\text{T}}\cdot R^{-1}(\rho )\cdot \text{stde}$$

where ***stde*** is the *N* × 1 vector with entries *stde*_*t*(*i*)_ = *e*_*t*(*i*)_/*σ*_*t*(*i*)_ for *t*(*i*) ∈ *T*. Consequently, ***g***(***β***′) has entries

$$g_{j}\left(\beta^{\prime }\right)=\text{stde}x_{j}^{\prime \text{T}}\cdot R^{-1}(\rho )\cdot \text{stde}-\sum_{i=1}^{N}x_{t(i),j}^{\prime }/2$$

for 1 ≤ *j* ≤ *J*′ where $\text{stde}x_{j}^{\prime }$ is the *N* × 1 vector with entries

$${\text{stdex}}_{t(i),j}^{\prime }=x_{t(i),j}^{\prime }\cdot {\text{stde}}_{t(i)}/2$$

for *t*(*i*) ∈ *T*. ***H***(***β***′) has entries

$$H_{j.j^{\prime }}\left(\beta^{\prime }\right)=-\text{stdex}x_{j.j^{\prime }}^{\prime \prime \text{T}}\cdot R^{-1}(\rho )\cdot \text{stde}-\text{stde}x_{j}^{\prime \text{T}}\cdot R^{-1}(\rho )\cdot \text{stde}x_{j^{\prime }}^{\prime }$$

for 1 ≤ *j*, *j*′ ≤ *J*′ where $\text{stde}x_{j}^{\prime }$ is the *N* × 1 vector with entries

$${\text{stdexx}}_{t(i)j,j^{\prime }}^{\prime \prime }=x_{t(i),j}^{\prime }\cdot x_{t(i),j^{\prime }}^{\prime }\cdot {\text{stde}}_{t(i)}/4$$

for *t*(*i*) ∈ *T*. ***H***(***β***, ***β***′) has columns

$$H_{j}\left(\beta ,\beta^{\prime }\right)=-D^{\text{T}}\cdot \text{Diag}\left(\sigma invx_{j}^{\prime }\right)\cdot R^{-1}(\rho )\cdot \text{stde}\;-D^{\text{T}}\cdot \text{Diag}(1/\sigma )\cdot R^{-1}(\rho )\cdot \text{stde}x_{j}^{\prime }$$

where $\sigma invx_{j}^{\prime }$ is the *N* × 1 vector with entries $\sigma invx_{t(i),j}^{\prime }=x_{t(i),j}^{\prime }/\left(2\cdot \sigma_{t(i)}\right)$ for *t*(*i*) ∈ *T* and 1 ≤ *j* ≤ *J*′. If offsets are included, they are carried along in equations without any effect on derivatives.

### 4.2. Parameter Estimation

Given a value for the vector ***θ*** of all coefficient parameters, an estimate of the correlation parameter *ρ* can be based on the associated standardized residuals *stde*_*t*(*i*)_. Calculate correlation estimates for the IND, EXCH, and AR1 correlation structures using the same formulas as before but computed with these more general standardized residuals. Iteratively solve ***g***(***θ***) = 0 as follows. Given the current value ***θ***_*u*_ for ***θ***, the next value is given by ***θ***_*u*+1_ = ***θ***_*u*_ − **H**^−1^ (***θ***_*u*_) · ***g***(***θ***_*u*_), thereby adapting Newton’s method with ***g***(***θ***) in the role of the gradient vector and **H**(***θ***) in the role of the Hessian matrix. The solution to the estimating equations for observations indexed by *T* is denoted as ***θ***(*T*) = (***β***(*T*)^T^ ***β**′*(*T*)^T^)^T^ with associated correlation estimate *ρ*(*T*) = *ρ*(***θ***(*T*)).

## 5. Extended Linear Mixed Modeling

### 5.1. Formulation

GEE modeling can be further extended to handle full parameter estimation through maximizing the likelihood-like function. Let ***θ*** = (***β***^T^ ***β***′^T^ *ρ*)^T^ be the (*J* + *J*′ + 1) × 1 vector of the mean, dispersion, and correlation parameters. The definition of the likelihood-like function *L*(*T*;***θ***) given for standard GEE holds using this more general parameter vector ***θ***. The likelihood-like function *L*(*T*;***θ***) is maximized in the coefficient parameter vector ***θ*** by solving the estimating equations

g(θ)=∂ℓ(T;θ)/∂θ=0

where ∂*ℓ*(*T*;***θ***)/∂***θ*** is the vector of standard partial derivatives of *ℓ*(*T*;***θ***). The associated matrix ***H***(***θ***) = ∂***g***(***θ***)∂***θ***. In this case, ***g***(***θ***) is a true gradient vector and ***H***(***θ***) a true Hessian matrix. This approach is extended linear mixed modeling in the sense that if the entries of ***y*** were continuous variables treated as normally distributed with *V*(*μ*) = 1, then it would be exactly linear mixed modeling. Formulations given in what follows are adapted from those of [8].

The gradient vector ***g***(***θ***) = (***g***(***β***)^T^ ***g***(***β***′)^T^ ***g***(*ρ*))^T^. The gradient sub-vector ***g***(***β***′) = ∂*ℓ*(*T*;***θ***)/∂***β***′ has the same formulation as for extended GEE modeling, only now its entries are standard partial derivatives. The gradient subvector ***g***(***β***) = ∂*ℓ*(*T*;***θ***)/∂***β*** has entries

$$g_{j}(\beta )=\text{stde}x_{j}^{\text{T}}\cdot R^{-1}(\rho )\cdot \text{stde}-\sum_{i=1}^{N}x_{t(i),j}/2$$

where ***stdex***_*j*_ is the *N* × 1 vector with entries

$${\text{stdex}}_{t(i),j}=x_{t(i),j}\left(y_{t(i)}+\mu_{t(i)}\right)/\left(2\cdot \sigma_{t(i)}\right)$$

for *t*(*i*) ∈ *T* and 1 ≤ *j* ≤ *J*. The partial derivative *g*(*ρ*) = ∂*ℓ*(*T*;***θ***)/∂*ρ* satisfies

$$g(\rho )=-{\text{stde}}^{\text{T}}\cdot \partial R^{-1}(\rho )/\partial \rho \cdot \text{stde}/2-\partial (\text{log}(|R(\rho )|))/\partial \rho /2$$

where

$$\partial (\text{log}(|R(\rho )|))/\partial \rho =tr\left(R^{-1}(\rho )\cdot \partial R(\rho )/\partial \rho \right),$$

*tr* denotes the trace function, and

$$\partial R^{-1}(\rho )/\partial \rho =-R^{-1}(\rho )\cdot \partial R(\rho )/\partial \rho \cdot R^{-1}(\rho ).$$

For IND correlations, ∂**R**(*ρ*)/∂*ρ* = **0**. For EXCH correlations, ∂**R**(*ρ*)/∂*ρ* is the *N* × *N* matrix with diagonal entries all equal to 0 and off-diagonal entries all equal to 1. For AR1 correlations, ∂**R**(*ρ*)/∂*ρ* is the *N* × *N* matrix with diagonal entries all equal to 0 and off-diagonal entries equaling

$$\left|t(i)-t\left(i^{\prime }\right)\right|\cdot \rho_{\text{AR}1}^{\left|t(i)-t\left(i^{\prime }\right)\right|-1}$$

in the *i*^*th*^ row and *i*′^*th*^ column for 1 ≤ *i* ≠ *i*′ ≤ *N*.

***H***(***β***) has nine component submatrices: the *J* × *J* matrix ***H***(***β***) = ∂***g***(***β***)/∂***β*** for the mean parameters, the *J* ′× *J* ′ matrix ***H***(***β***′) = ∂***g***(***β***′)/∂***β***′ for the dispersion parameters computed as for extended GEE modeling, the second partial derivative *H*(*ρ*) = ∂*g*(*ρ*)/∂*ρ* for the correlation parameter, the *J* × *J*′ matrix ***H***(***β***, ***β***′) = ∂***g***(***β***)/∂***β***′, and its transpose ***H***(***β, β′***) = ***H***(***β, β′***)^T^, the *J* × 1 vector ***H***(***β***, *ρ*) = ∂***g***(***β***)/∂_*ρ*_ and its transpose ***H***(*ρ*, ***β***) = ***H***(***β***, *ρ*)^T,^ and the *J*′ × 1 vector ***H***(***β***′, *ρ*) = ∂***g***(***β***′)/∂_*ρ*_ and its transpose ***H***(*ρ*, ***β***′) = ***H***(***β***′,*ρ*)^T^. ***H***(***β***) has entries

$$H_{j,j^{\prime }}(\beta )=-\text{stdex}x_{j,j^{\prime }}^{\text{T}}\cdot R^{-1}(\rho )\cdot \text{stde}-\text{stde}x_{j}^{\text{T}}\cdot R^{-1}(\rho )\cdot \text{stde}x_{j^{\prime }}$$

for 1 ≤ *j*, *j*′ ≤ *J*′ where ***stdexx***_*j, j′*_ is the *N* × 1 vector with entries

$${\text{stdexx}}_{t(i)j,j^{\prime }}=x_{t(i),j}\cdot x_{t(i),j^{\prime }}\cdot {\text{stde}}_{t(i)}/4$$

for *t*(*i*) ∈ *T*. The second partial derivative *H* (*ρ*) satisfies

$$H(\rho )=-{\text{stde}}^{\text{T}}\cdot \partial^{2}R^{-1}(\rho )/\partial \rho^{2}\cdot \text{stde}/2-\partial^{2}(\text{log}(|R(\rho )|))/\partial \rho^{2}/2$$

where

$$\begin{array}{l} \partial^{2}(\text{log}(|R(\rho )|))/\partial \rho^{2}=-tr\left(R^{-1}(\rho )\cdot \partial R(\rho )/\partial \rho \cdot R^{-1}(\rho )\cdot \partial R(\rho )/\partial \rho \right)\\ +tr\left(R{\left(\rho \right)}^{-1}\cdot \partial^{2}R(\rho )/\partial \rho^{2}\right). \end{array}$$

For IND and EXCH correlations, ∂^2^**R**(*ρ*)/∂*ρ*^2^ = 0. For AR1 correlations, ∂^2^**R**(*ρ*)/∂*ρ*^2^ is the *N* × *N* matrix with diagonal entries all equal to 0 and off-diagonal entries equaling

$$\left|t(i)-t\left(i^{\prime }\right)\right|\cdot \left(\left|t(i)-t\left(i^{\prime }\right)\right|-1\right)\cdot \rho_{\text{AR}1}^{\left|t(i)-t\left(i^{\prime }\right)\right|-2}$$

in the *i*^*th*^ row and *i*′^*th*^ column for 1 ≤ *i* ≠ *i*′ ≤ *N*. ***H***(***β***′, ***β***) has entries

$$H_{j,j^{\prime }}\left(\beta^{\prime },\beta \right)=-\text{stdex}x_{j,j^{\prime }}^{\prime \text{T}}\cdot R^{-1}(\rho )\cdot \text{stde}-\text{stde}x_{j}^{\text{T}}\cdot R^{-1}(\rho )\cdot \text{stde}x_{j^{\prime }}^{\prime }$$

for 1 ≤ *j, j*′ ≤ *J*′ where $\text{stdex}x_{j,j^{\prime }}^{\prime }$ is the *N* × 1 vector with entries

$${\text{stdexx}}_{t(i)j,j^{\prime }}^{\prime }=x_{t(i),j^{\prime }}^{\prime }\cdot {\text{stdex}}_{t(i),j}/2$$

for *t*(*i*) ∈ *T*, 1 ≤ *j* ≤ *J*, and 1 ≤ *j*′ ≤ *J*′. ***H***(***β***, *ρ*) has entries

$$H_{j}(\beta ,\rho )=\text{stde}x_{j}^{\text{T}}\cdot \partial R{\left(\rho \right)}^{-1}/\partial \rho \cdot \text{stde}$$

for 1 ≤ *j ≤ J*. ***H***(***β***′, *ρ*) has entries

$$H_{j}\left(\beta^{\prime },\rho \right)=\text{stde}x_{j}^{\prime \text{T}}\cdot \partial R^{-1}(\rho )/\partial \rho \cdot \text{stde}$$

for 1 ≤ *j* ≤ *J*′.

### 5.2. Parameter Estimation

The parameter vector ***θ*** is estimated by iteratively solving ***g***(***θ***) = 0 as follows. Given the current value ***θ***_*u*_ for ***θ***, the next value is given by

$$\theta_{u+1}=\theta_{u}-H^{-1}\left(\theta_{u}\right)\cdot g\left(\theta_{u}\right),$$

thereby using Newton’s method with gradient vector ***g***(***θ***) and Hessian matrix **H**(***θ***). The estimation process can be stopped early if *ℓ*(*T*;***θ***_*u*+1_) does not increase by much compared to *ℓ*(*T*;***θ***_*u*)_. The solution to the estimating equations T for observations indexed by *T* is denoted as $\theta (T)={\left(\beta {\left(T\right)}^{\text{T}}\beta^{\prime }{\left(T\right)}^{\text{T}}\rho (T)\right)}^{\text{T}}$.

The covariance matrix for the parameter estimate vector ***θ***(*T*) can be computed as −**H**^−1^ (***θ***(*T*)) and the variances corresponding to its diagonal entries can be used to compute *z* tests of zero individual model parameters. These are useful for fixed models of theoretical importance. On the other hand, tests for parameters of adaptively generated models (as described in Section 6) are usually significant as a consequence of the model selection process, and so results for these tests are not reported for models generated in the example analyses.

## 6. Modeling Possibly Nonlinear Means and Dispersion over Time

Knafl and Ding [3] provide a detailed formulation for adaptively searching through alternative regression models for means and dispersions in a variety of contexts using adaptive fractional polynomial models [9]. A brief overview is provided here. These methods are used in the example analyses of individual cancer patient count/rate data presented later. Model selection proceeds through two phases. The expansion phase first grows the model adding in alternative power transforms of predictors for means and dispersions. The contraction phase then reduces the model to a parsimonious set of power transforms by removing transforms from the current model one at a time and adjusting the powers of the remaining transforms. Alternative models are evaluated using LCV scores. The modeling process is controlled by tolerance parameters indicating how much of a reduction in the LCV score can be tolerated at given stages of the process. Knafl and Ding [3] also provide a wide variety of example analyses demonstrating the usefulness of these adaptive regression methods. A description of these methods in the standard Poisson regression context is provided in [10].

A SAS^®^ (SAS Institute, Inc., Cary, NC) macro has been developed for generating adaptive analyses including the reported example analyses. This macro as well as data and code used to generate the results of the example analyses are available from the first author.

## 7. Example Analyses

### 7.1. Pain Flare Counts per Day

**Figure 1.**
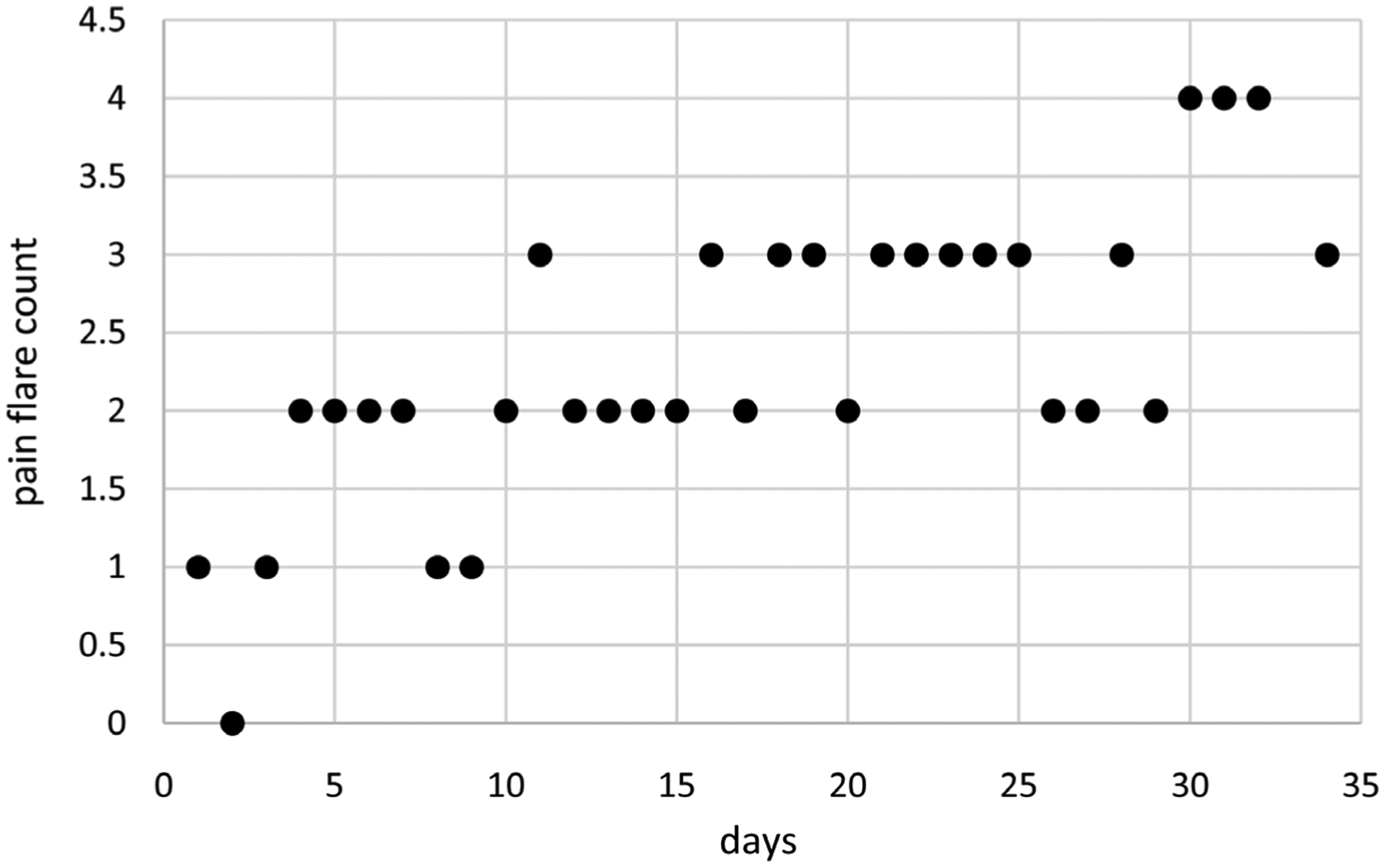
Pain flare counts over time for cancer patient 1.

Figure 1 displays pain flare counts for Cancer Patient 1 over a period of 34 days. Pain flares range from 0 to 4 per day and tend to increase over time. Data are available for *N* = 33 days with a missing value for one day (day 33). These data were collected using Ecological Momentary Assessment (EMA) [11] as implemented in the mEMA app [12].

Table 1 contains results for adaptive models for means and dispersions of pain flare counts over time using the two modeling approaches extended GEE modeling and extended linear mixed modeling and the three correlation structures IND, AR1, and EXCH. Power transforms reported in Table 1 were generated by adaptively searching through alternative power transforms using the methods described in Section 6. LCV scores are based on *k* = 5 folds with fold sizes ranging from 2 to 8 measurements and no empty folds. For extended GEE modeling, IND correlations generate the best LCV score 0.38018 over the three correlation structures. For extended linear mixed modeling, IND correlations also generate the best LCV score 0.40622. These results suggest that a Poisson process assumption is reasonable for these pain flare counts.

**Figure 2.**
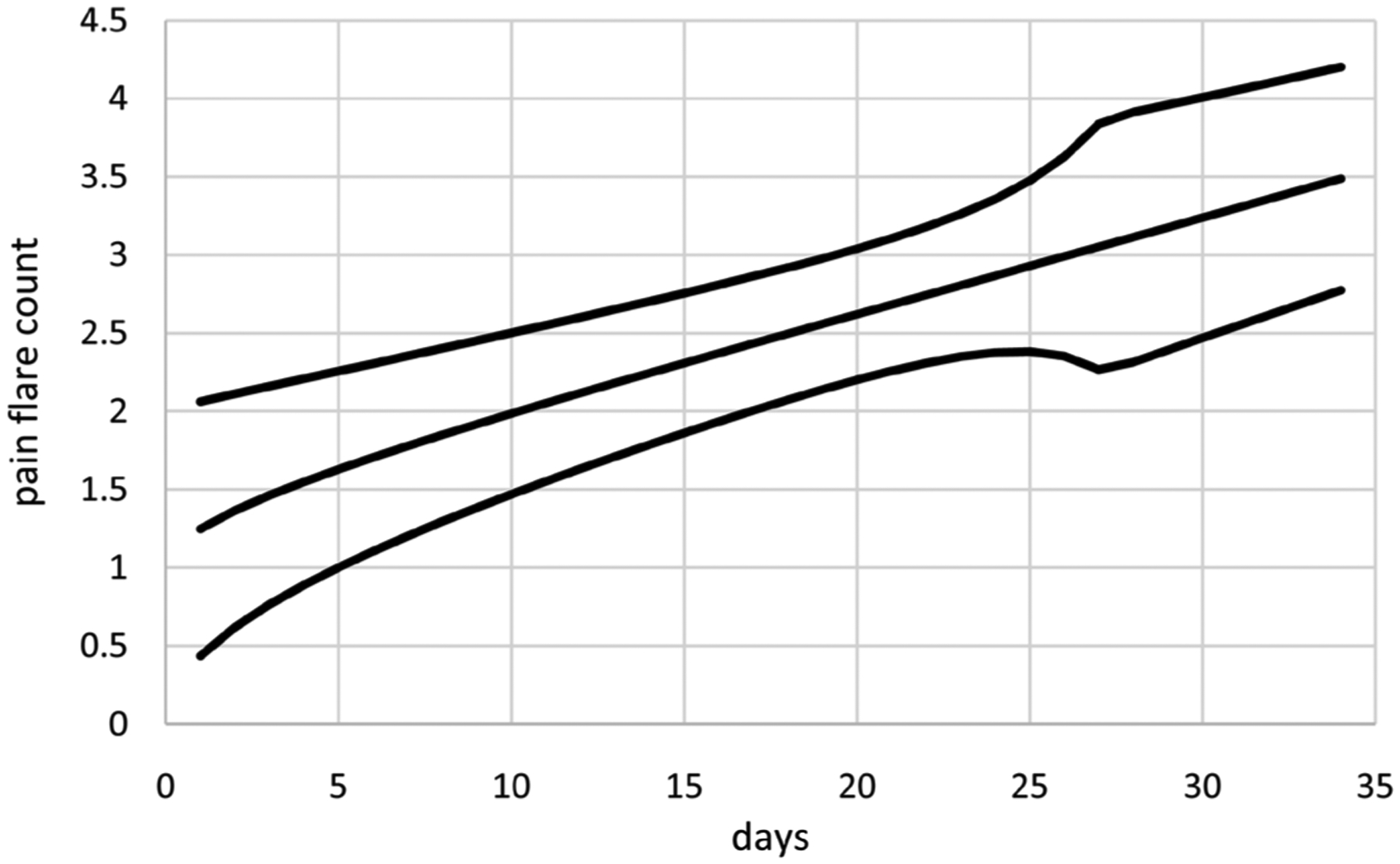
Mean pain flare counts (middle curve) with unit error bounds for cancer patient 1.

**Figure 3.**
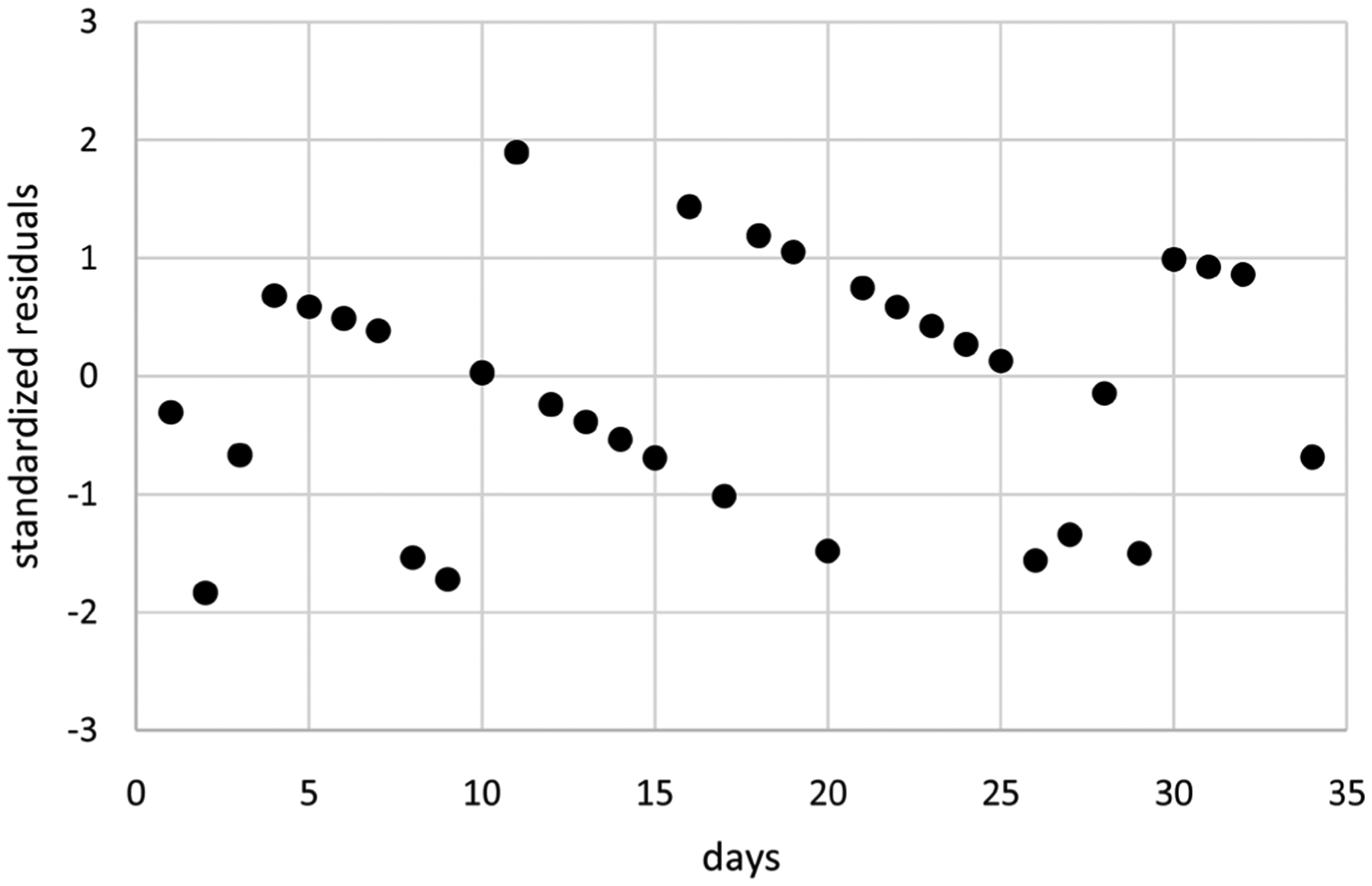
Standardized residuals for the model of Figure 2.

Extended linear mixed modeling generates better LCV scores than extended GEE for all three correlation structures. Moreover, computation times are much shorter ranging from 0.4 to 1.2 minutes compared to 13.9 to 35.5 minutes. These results suggest that extended linear mixed modeling is preferable for modeling these pain flare counts because it generates better LCV scores in less time. Consequently, only extended linear mixed modeling using IND correlations is considered further for these data, generating the model with means based on *t*(*i*)^0.49^ without an intercept and dispersions based on *t*(*i*)^8.37^ and *t*(*i*)^0.5^ without an intercept. Figure 2 displays estimates of mean pain flare counts over time along with unit error bands over time (*i.e*., the mean ±1 extended standard deviation at each time) to account for variability about the means. Mean pain flare counts increase over time, somewhat close to linearly in time. Variability in pain flare counts is smaller in the middle of the period, somewhat larger at the start of the period and even larger at the end of the period. Figure 3 displays standardized residuals for this model, which range between ±2 without any extreme outliers, suggesting the model is a reasonable fit for these data.

The associated model generated using *k* = 10 folds has similar means based on *t*(*i*)^0.5^ without an intercept and simpler dispersions based on *t*(*i*)^0.2^ without an intercept. However, the 10-fold LCV score 0.38107 is smaller, suggesting that *k* = 5 is a better choice for these data. Moreover, there is one empty fold, suggesting that the choice of *k* = 10 folds is too large for these data with only *N* = 33 measurements. The associated model generated with *k* = 5 folds and assuming constant dispersions has a similar model for the means based on *t*(*i*)^0.53^ without an intercept but a smaller LCV score 0.37031, suggesting that the dispersions for these data are reasonably treated as nonconstant over time.

### 7.2. As Needed Pain Medications Taken Counts per Day

**Figure 4.**
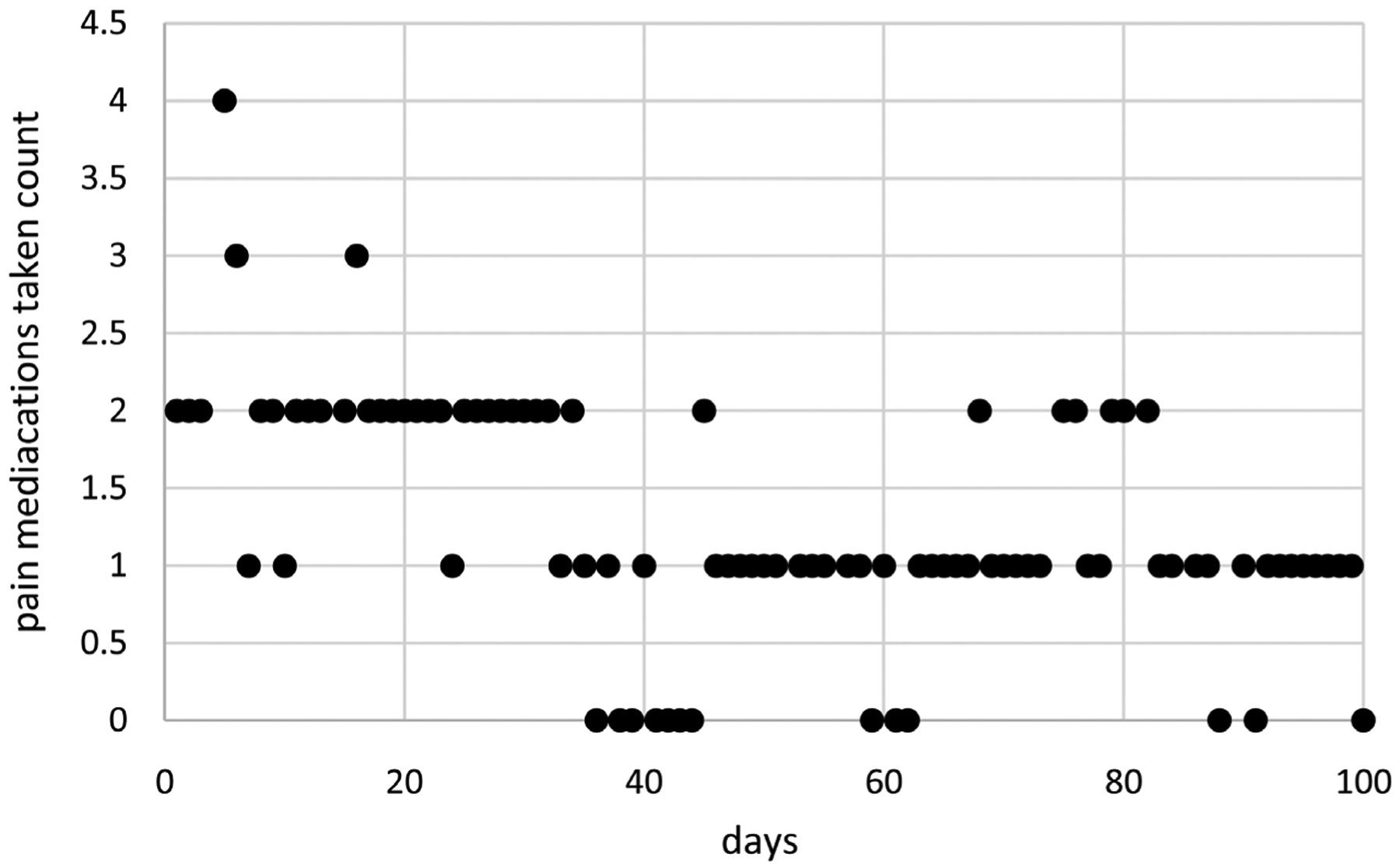
As needed pain medications taken counts over time for cancer patient 2.

Figure 4 displays as needed pain medications taken counts for Cancer Patient 2 over a period of 100 days. As needed pain medications taken counts range from 0 to 4 per day and tend to decrease over time. Data are available for *N* = 92 days with a missing value for eight other days (days 4, 14, 52, 56, 74, 81, 85, and 89). These data were also collected using the mEMA app.

Table 2 contains results for adaptive models for means and dispersions of as needed pain medications taken counts over time using the two modeling approaches extended GEE modeling and extended linear mixed modeling and the three correlation structures IND, AR1, and EXCH. LCV scores are based on *k* = 5 folds with fold sizes ranging from 13 to 21 measurements with no empty folds. For extended GEE modeling, AR1 correlations generate the best LCV score 0.41497 over the three correlation structures. For extended linear mixed modeling, AR1 correlations generate the best LCV score 0.40509. These results indicate that a Poisson process assumption may not be appropriate for these as needed pain medications taken counts.

**Figure 5.**
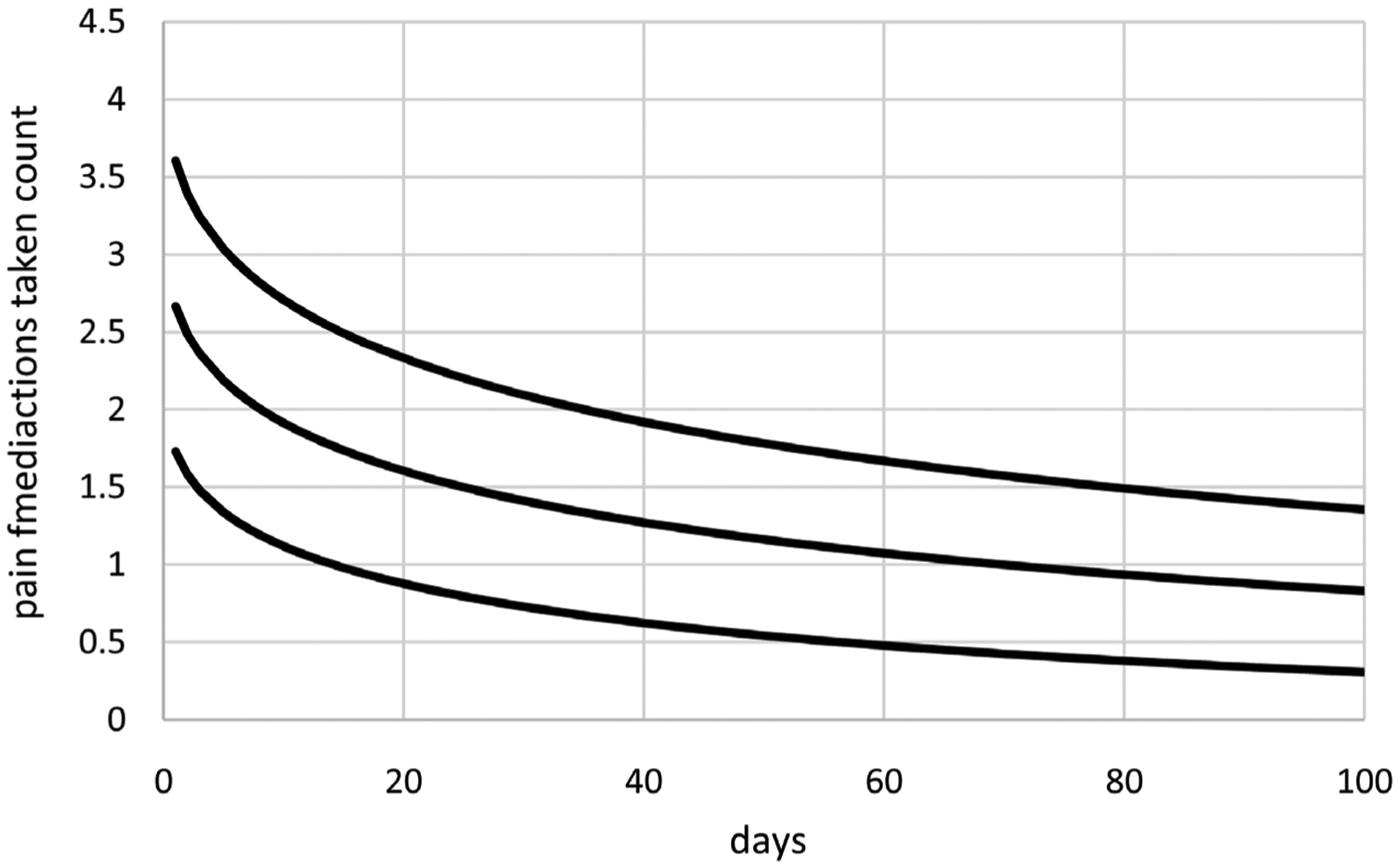
Mean as needed pain medications taken counts (middle curve) with unit error bounds for cancer patient 2.

**Figure 6.**
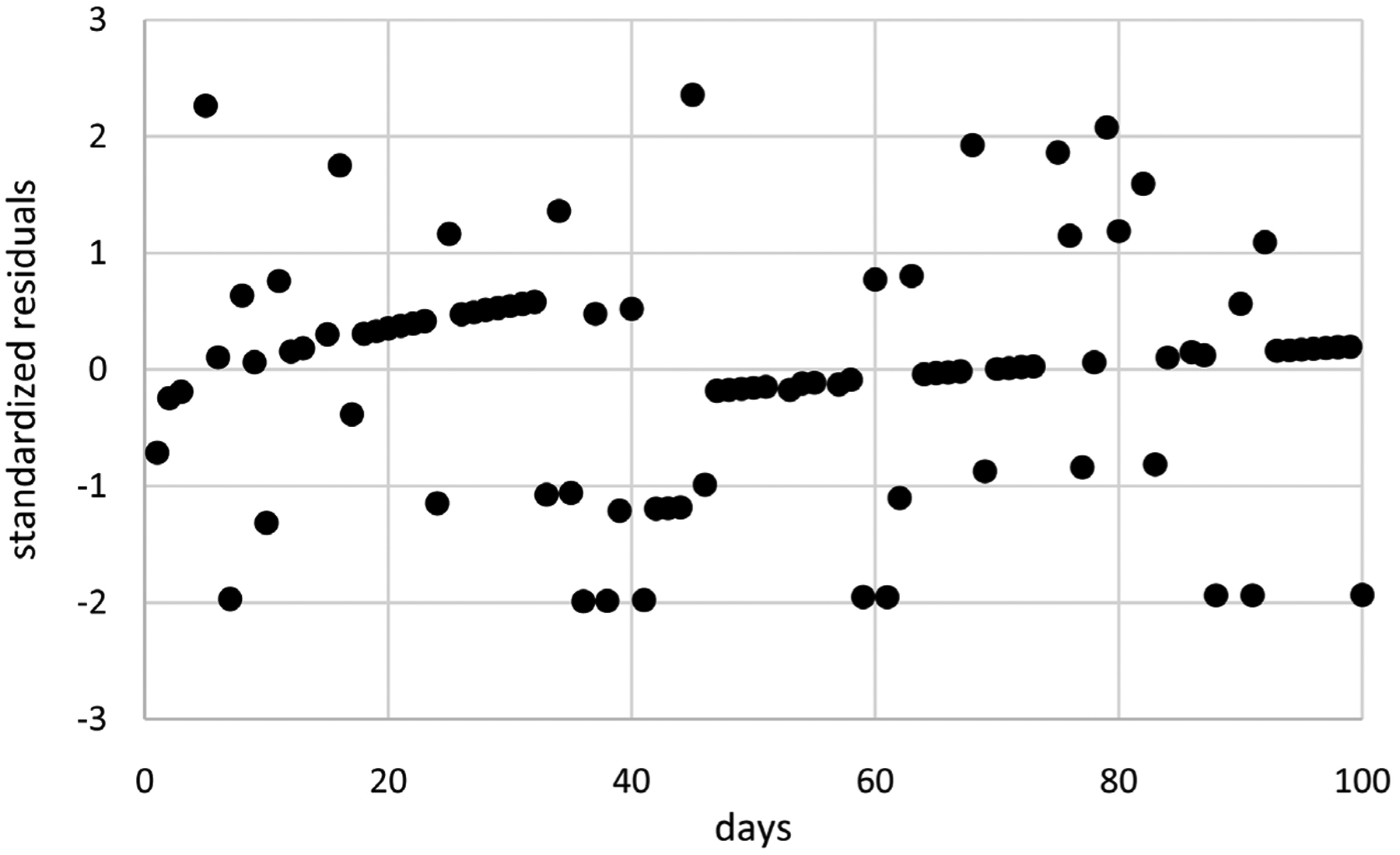
Standardized residuals for the model of Figure 5.

Extended GEE modeling generates a better LCV score than extended linear mixed modeling for the IND correlation structure, but the scores for these two approaches are not too different. Extended linear mixed modeling generates a better LCV score than extended GEE modeling for the EXCH correlation structure. Extended GEE modeling generates a better LCV score than extended linear mixed modeling for the AR1 correlation structure. Although this is the best overall LCV score, the associated model for extended linear mixed modeling is more parsimonious with an intercept and one time transform for the means compared to two time transforms and constant dispersions compared to dispersions based on and intercept and one time transform. Moreover, computation times are substantially shorter for extended linear mixed modeling ranging from 0.5 to 1.7 minutes compared to 84.5 to 222.7 minutes or 1.4 to 3.7 hours. These results suggest that extended linear mixed modeling is preferable for modeling these as needed pain medications taken counts because it generates competitive or better scores or more parsimonious models in substantially less time. Consequently, only extended linear mixed modeling using AR1 correlations are considered further for these data, generating the model with means based on *t*(*i*)^0.4^ with an intercept, constant dispersions based on an intercept, and estimated autocorrelation *ρ*_AR1_ = 0.45. Figure 5 displays estimates of mean as needed pain medications taken counts over time along with unit error bands over time (i.e., the mean ±1 extended standard deviation at each time) to account for variability about the means. Mean as needed pain medications taken counts decrease nonlinearly over time. Variability in as needed pain medications taken counts is close to constant over time. Figure 6 displays standardized residuals for this model, which range well within ±3 without any extreme outliers, suggesting the model provides a reasonable fit to these data.

The associated model generated using k = 10 folds is about the same with means based on *t*(*i*)^0.5^ with an intercept, constant dispersions, and estimated correlation *ρ*_AR1_ = 0.45. The 10-fold LCV score 0.40958 is larger, suggesting that *k* = 10 is a better choice for these data. There are no empty folds. The associated model generated using *k* = 15 folds is similar with means based on *t*(*i*)^0.5^ with an intercept, dispersions based on *t*(*i*)^0.07^ without an intercept, and estimated correlation *ρ*_AR1_ = 0.46. The 15-fold LCV score 0.40353 is smaller, suggesting that *k* = 10 is a better choice for these data. There are no empty folds. The associated model generated with *k* = 15 folds assuming constant dispersions has means based on *t*(*i*)^0.5^ with an intercept and close 15-fold LCV score 0.40318. Consequently, models generated by 5, 10, and 15 folds using extended linear mixed modeling are not too different, suggesting that the results are reasonably robust to the choice of the number of folds.

### 7.3. Around the Clock Pain Medications Taken Rates per Day per Dose

**Figure 7.**
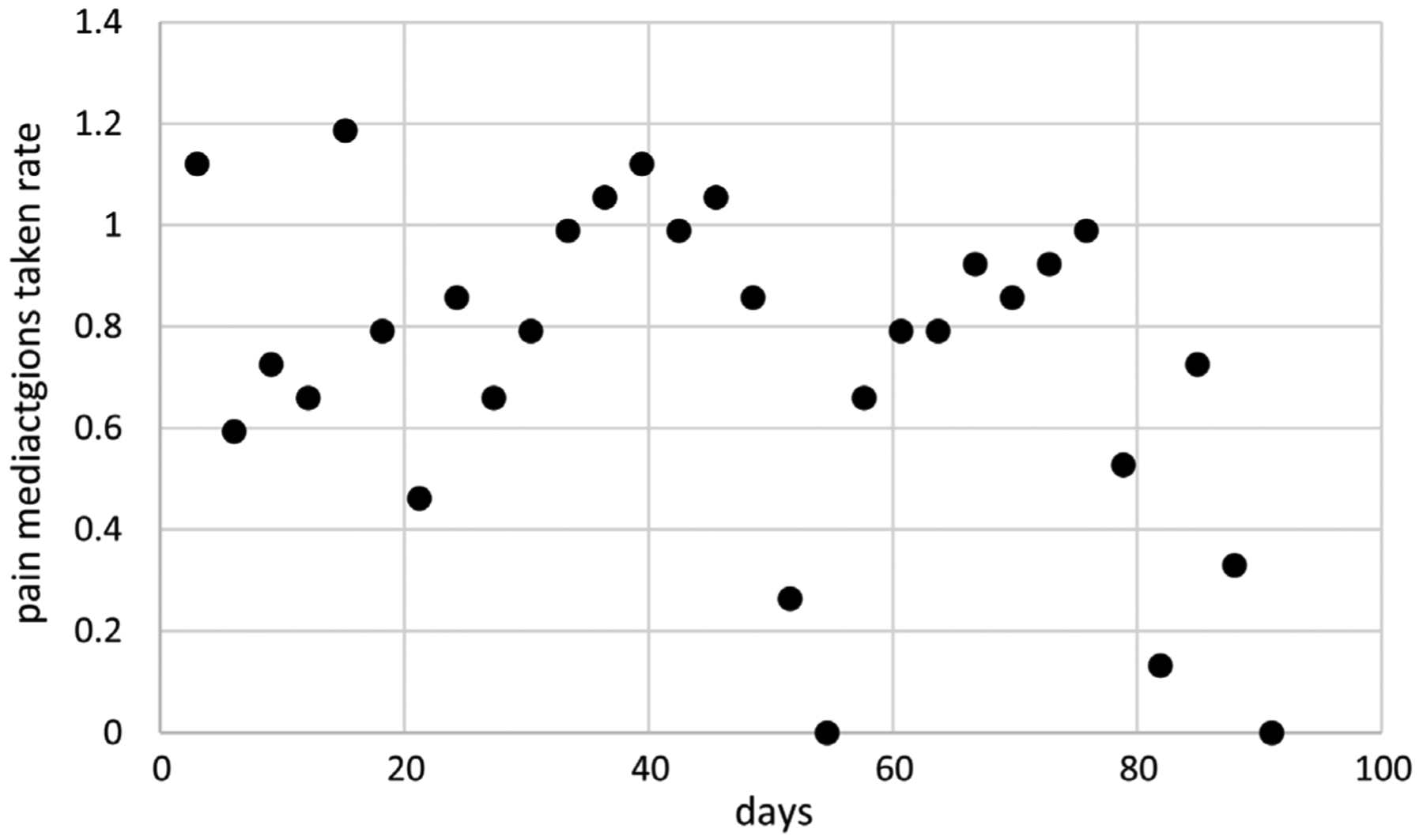
Around the clock pain medications taken rates per day per dose over time for cancer patient 3.

Adherence data for around the clock pain medications were collected using pill bottles equipped with Medication Event Monitoring System (MEMS) devices (AARDEX North America, Boulder, CO) that recorded the date and time of each pill bottle opening and presumably of the taking of the pain medication [13] [14]. Cancer Patient 3 was monitored for a period of 91 days. Counts of around the clock pain medications taken were computed for 30 equal-sized subperiods of 3.03 days each, ranging from 0 to 18. Around the clock pain medications were to be taken five times a day by this patient. Methods for modeling such data assuming the special case of a Poisson process with constant dispersions are provided in [10]. Figure 7 displays around the clock pain medications taken rates per day per dose for Cancer Patient 3. The ideal rate of 1 means that the patient took around the clock pain medications at the appropriate rate over the associated time subperiod. Around the clock pain medications taken rates range from 0 to 1.19 per day per dose and tend to decrease over time. Data are available for *N* = 30 subperiods with none missing.

Table 3 contains results for adaptive models for means and dispersions of around the clock pain medications taken rates over time using the two modeling approaches extended GEE modeling and extended linear mixed modeling and the three correlation structures IND, AR1, and EXCH. LCV scores are based on *k* = 5 folds with fold sizes ranging from 2 to 8 measurements with no empty folds. For extended GEE modeling, EXCH correlations generate the best LCV score 0.051583 over the three correlation structures. For extended linear mixed modeling, AR1 correlations generate the best LCV score 0.053856, which is also the best overall LCV score for Table 3 models. For both modeling approaches, the LCV score for IND correlations is quite a bit smaller than the best LCV score over the three correlation structures. These results indicate that a Poisson process assumption may not be appropriate for these around the clock pain medications taken rates.

**Figure 8.**
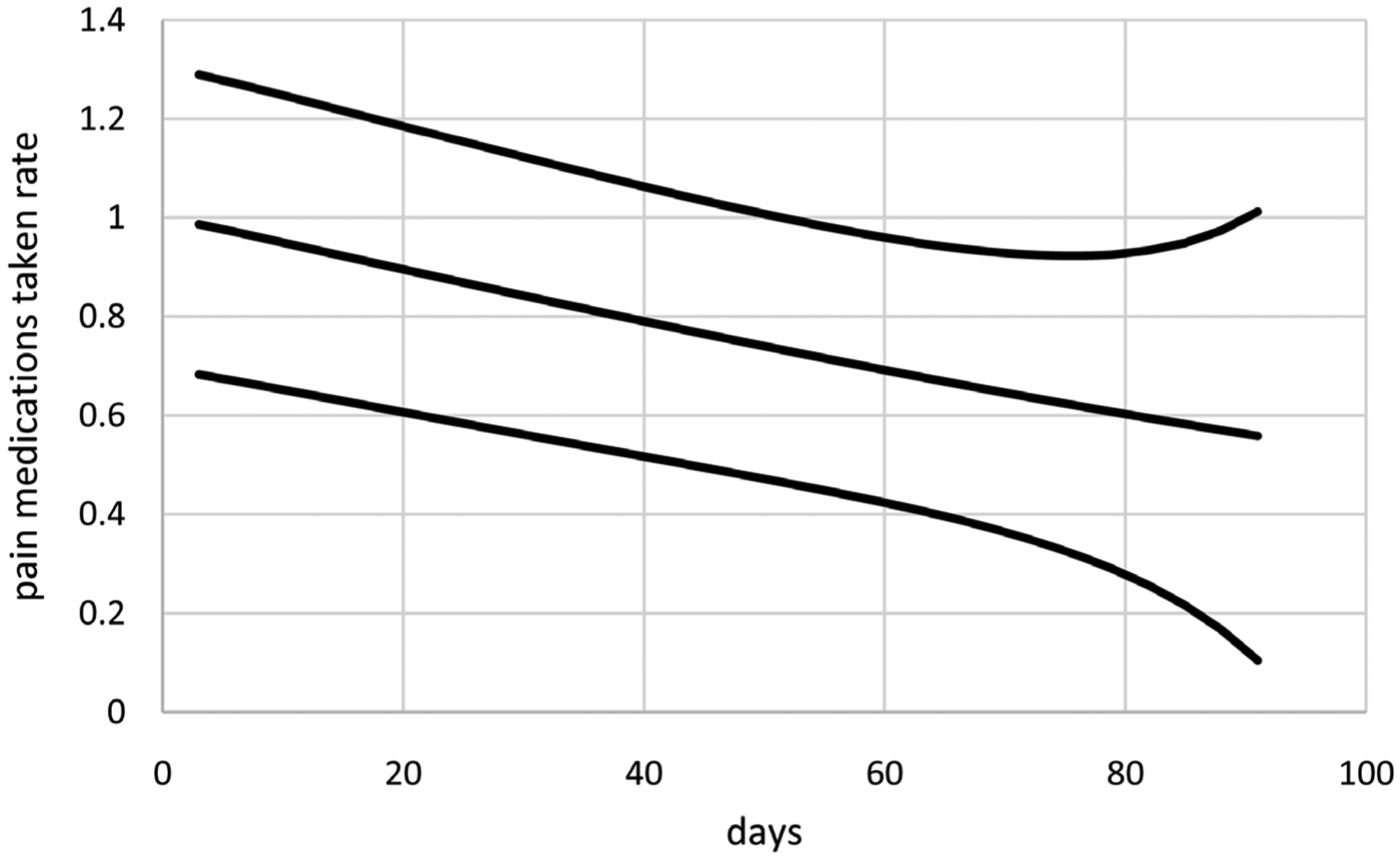
Mean around the clock pain medications taken rates per day per dose (middle curve) with unit error bounds for cancer patient 3.

**Figure 9.**
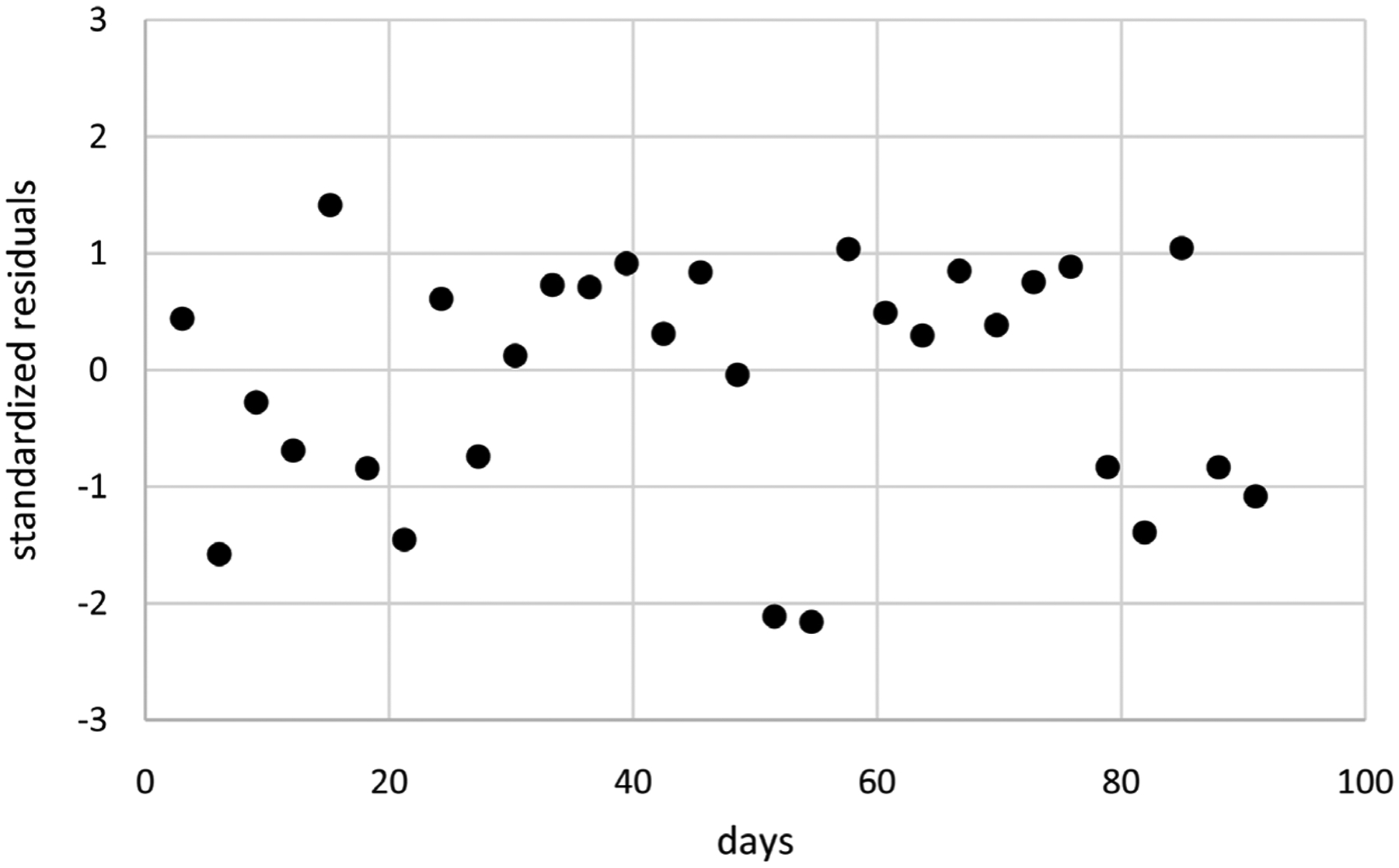
Standardized residuals for the model of Figure 8.

Extended linear mixed modeling generates better LCV scores than extended GEE for the IND and AR1 correlation structures. Its LCV score is smaller for the EXCH correlation structure, but its model is more parsimonious based on one time transform for the means with constant dispersions compared to one time transform plus an intercept for the means with constant dispersions. Furthermore, computation times are much shorter for extended linear mixed modeling ranging from 0.2 to 0.7 minutes compared to 5.1 to 11.9 minutes. These results suggest that extended linear mixed modeling is preferable for modeling these around the clock pain medications taken rates because it generates the best LCV score in less time. Consequently, only extended linear mixed modeling using AR1 correlations are considered further for these data, generating the model with means based on *t*(*i*)^1.1^ without an intercept, dispersions based on *t*(*i*)^6.1^ with an intercept, and estimated autocorrelation *ρ*_AR1_ = 0.75. Figure 8 displays estimates of mean around the clock pain medications taken rates over time along with unit error bands over time (*i.e*., the mean ±1 extended standard deviation at each time) to account for variability about the means. Mean around the clock pain medications taken counts decrease close to linearly over time. Variability in around the clock pain medications taken rates is larger at the end of the period. Figure 9 displays standardized residuals for this model, which range well within ±3 without any extreme outliers, suggesting the model provides a reasonable fit to these data.

The associated model generated using *k* = 10 folds is somewhat similar with means based on *t*(*i*)^0.4^ without an intercept, constant dispersions based on an intercept, and estimated autocorrelation *ρ*_AR1_ = 0.76. However, the 10-fold LCV score 0.052023 is smaller, suggesting that *k* = 5 is a better choice for these data. Moreover, there is one empty fold, suggesting that the choice of *k* = 10 folds is too large for these data with only *N* = 30 measurements. The associated model generated with *k* = 5 folds and assuming constant dispersions has a model for the means based on based on *t*(*i*)^1.01^ without an intercept, an autocorrelation estimate of *ρ*_AR1_ = 0.75, and a smaller LCV score 0.0.050386, suggesting that the dispersions for these data are reasonably treated as nonconstant over time.

## 8. Discussion

### 8.1. Summary

Methods are formulated for modeling individual patient count/rate data over time allowing for nonlinear trajectories for means, time-varying dispersions, and temporal correlation. Three correlation structures are considered including IND, EXCH, and spatial AR1 correlations. Two extensions of standard GEE modeling are considered. Extended GEE modeling augments standard GEE mean parameter estimating equations with dispersion parameter estimating equations while using the GEE approach for correlation parameter estimation. Extended linear mixed modeling estimates all model parameters using estimating equations for mean, dispersion, and correlation parameters. These new estimating equations are determined by partial derivatives of a likelihood-like function based on the multivariate normal density. This likelihood-like function is also used to define a likelihood-like cross-validation (LCV) score for evaluating models. LCV scores are used to control adaptive regression modeling of possibly nonlinear means and dispersions over time. It is also possible to generate penalized likelihood-like criteria for model selection generalizing standard penalized likelihood criteria [15] such as the commonly used Akaike information criterion (AIC) and Bayesian information criterion (BIC). Pan [16] has formulated a penalized model selection criterion related to the AIC called the quasi-likelihood information criterion (QIC) for GEE model selection, but the QIC score does not fully account for the correlation structure. Model selection criteria based on the likelihood-like function fully account for the correlation structure.

Example analyses using these methods are provided using three types of count/rate data for individual cancer patients including cancer pain flares per day, as needed cancer pain medications taken per day, and around the clock cancer pain medications taken per day per dose. Extended linear mixed modeling generates models with either better LCV scores or more parsimonious models than extended GEE modeling. Moreover, times to compute models are substantially smaller for extended linear mixed modeling than for extended GEE modeling. Time differences can be extreme for even moderate samples sizes, for example, analyses for the second example data set with 92 observations required at most 1.7 minutes for extended linear mixed modeling compared to up to 3.7 hours for extended GEE. These results indicate that extended linear mixed modeling is preferable for modeling individual patient count/rate data over time. This is likely to hold in more general modeling situations with other types of data and for combined data for multiple patients.

### 8.2. Alternative Approaches

The formulation provided here assumes that separate modeling of each patient’s longitudinal data is preferable to modeling the combined data for all patients. Separate modeling is a person-centered approach to modeling longitudinal data as opposed to a variable-centered approach using the combined data [17] [18]. This is only feasible when there are substantial numbers of time measurements for each patient. Modeling the combined data for all patients typically involves the assumption that means and dispersions for all patients are reasonably treated as having the same functional form. Knafl *et al*. [10] provide an example where this is not an appropriate assumption for a specific set of data on medication taken rates per day for HIV patients on antiretroviral medications. In any case, the methods considered here generalize to handle combined data for multiple patients, not only count/rate longitudinal data but also continuous and dichotomous longitudinal data, and not just data for cancer patients.

Multilevel (or hierarchical linear) modeling [19] [20] could alternatively be used to provide for individual patient differences, but that usually accounts for nonlinearity using polynomial models, often simple quadratic models. Polynomial models can be too simplistic for addressing general nonlinearity. Knafl and Ding [3] provide an example for independent data where the polynomial model generating the best LCV score for degrees 0–3 is the degree 0 constant model, but a nonlinear adaptive regression model generates a much better LCV score. Future research is needed to investigate general nonlinearity using adaptive regression methods applied to multilevel models as well as to random effects models [21] and to generalized linear mixed models [22].

Spatial AR1 correlations generate better models than independent and exchangeable correlations for two of the three example data sets. This suggests consideration of autoregressive and/or moving average correlations [23] of orders more than 1. As the number of time points increases, even relatively large autocorrelations can generate small correlations for larger distances apart. For example, the third example data set had an estimated autocorrelation of *ρ*_AR1_ = 0.75 with integer time measurements ranging from 1 to 30 so that the smallest correlation is 0.75^29^ = 0.0002. The second example data set had an even smaller estimated autocorrelation of *ρ*_AR1_ = 0.45 with an even larger range of 1 to 100 integer time measurements so that the smallest correlation is 0.45^99^ = 4.7 × 10^−35^.These results suggest consideration of banded correlation autoregressive structures with zero correlations for measurements further apart than some fixed amount.

## Figures and Tables

**Table 1.** Adaptive models for means and dispersions of pain flare counts over time for alternative modeling approaches and correlation structures.

Modeling Approach	Correlation		Power Transforms^a^		5-fold LCV Score	Time^b^
	Structure	Estimate	Means	Dispersions		
	IND	0	*t*(*i*)^0.54^	*t*(*i*)^0.12^	038018	13.9
extended GEE	EXCH	0.001	*t*(*i*)^0.869^	*t*(*i*)^0.29^	0.34712	35.5
	AR1	0.10	*t*(*i*)^0.59^	*t*(*i*)^0.1^	0.37404	17.6
	IND	0	*t*(*i*)^0.49^	*t*(*i*)^8.37^, *t*(*i*)^0.5^	0.40622	0.4
extended LMM	EXCH	0.42	*t*(*i*)^0.511^	*t*(*i*)^0.2^	0.36590	1.2
	AR1	0.20	*t*(*i*)^0.4^	1, *t*(*i*)^1.01^, *t*(*i*)^1.01^	0.37693	0.4

AR1—autoregressive of order 1; EXCH—exchangeable; GEE—generalized estimating equations; IND—independent; LCV—likelihood-like cross-validation; LMM—linear mixed modeling.

a. The *i*^th^ time value is denoted as *t*(*i*). A 1 corresponds to an intercept parameter; otherwise, the model has a zero intercept.

b. Difference in minutes of clock times between the start and end of computations.

**Table 2.** Adaptive models for means and dispersions of as needed pain medications taken counts over time for alternative modeling approaches and correlation structures.

Modeling Approach	Correlation		Power Transforms^a^		5-fold LCV Score	Time^b^
	Structure	Estimate	Means	Dispersions		
	IND	0	1, *t*(*i*)^0.5^	1	037030	84.5
extended GEE	EXCH	−0.01	1, *t*(*i*)^0.5^	1	0.36340	202.3
	AR1	0.57	*t*(*i*)^−0.12^, *t*(*i*)^3^	1, *t*(*i*)^−1.5^	0.41497	222.7
	IND	0	*t*(*i*)^0.3^, *t*(*i*)^0.1^	1	0.36845	0.5
extended LMM	EXCH	−0.01	1, *t*(*i*)^0.4^	1	0.37379	1.7
	AR1	0.45	1, *t*(*i*)^0.4^	1	0.40509	0.8

AR1—autoregressive of order 1; EXCH—exchangeable; GEE—generalized estimating equations; IND—independent; LCV—likelihood-like cross-validation; LMM—linear mixed modeling.

a. The *i*^th^ time value is denoted as *t*(*i*). A 1 corresponds to an intercept parameter; otherwise, the model has a zero intercept.

b. Difference in minutes of clock times between the start and end of computations.

**Table 3.** Adaptive models for means and dispersions of around the clock pain medications taken rates per day per dose over time for alternative modeling approaches and correlation structures.

Modeling Approach	Correlation		Power Transforms^a^		5-fold LCV Score	Time^b^
	Structure	Estimate	Means	Dispersions		
	IND	0	*t*(*i*)^0.7^	1	0.046556	5.1
extended GEE	EXCH	−0.03	1, *t*(*i*)^5^	1	0.051583	11.9
	AR1	0.58	*t*(*i*)^1.1^	1	0.048525	6.9
	IND	0	*t*(*i*)^0.8^	1	0.046837	0.2
extended LMM	EXCH	−0.03	*t*(*i*)^0.9^	1	0.045251	0.7
	AR1	0.75	*t*(*i*)^1.1^	1, *t*(*i*)^−1.5^	0.053856	0.2

AR1—autoregressive of order 1; EXCH—exchangeable; GEE—generalized estimating equations; IND—independent; LCV—likelihood-like cross-validation; LMM—linear mixed modeling.

a. The *i*^th^ time value is denoted as *t*(*i*). A 1 corresponds to an intercept parameter; otherwise, the model has a zero intercept.

b. Difference in minutes of clock times between the start and end of computations.
